# A novel integrated inflammatory-metabolic indicator as a potential predictor of obstructive sleep apnea: evidence from a clinical cohort and validation in the US National Health and Nutrition Examination Survey

**DOI:** 10.3389/fneur.2026.1813862

**Published:** 2026-04-10

**Authors:** Hengkang He, Jing Luo, Yixi Xiao, Xiong Zhang, Jingwen Zhang, Yang Tian, Jianhui Zhang

**Affiliations:** 1 Department of Otolaryngology Head and Neck Surgery, The Third People’s Hospital of Chengdu, College of Medicine, Southwest Jiaotong University, Chengdu, Sichuan, China; 2 Department of Otolaryngology Head and Neck Surgery, The Third People's Hospital of Chengdu, Chengdu, Sichuan, China

**Keywords:** AIP, inflammatory-metabolic composite indices, NHANES, OSA, UHR

## Abstract

**Background:**

Obstructive sleep apnea (OSA) is a prevalent sleep-disordered breathing condition closely associated with cardiovascular and metabolic risks. The current diagnostic gold standard, polysomnography, faces accessibility limitations, necessitating the development of simplified screening tools. Integrating multi-pathway information from hematological biomarkers may offer novel approaches.

**Objective:**

To systematically evaluate the association between multiple novel inflammatory-metabolic composite indices and OSA risk, and validate their stability across different populations.

**Methods:**

A two-stage cross-sectional study design was employed. Stage one utilized a clinical cohort from Chengdu Third People’s Hospital, China (*n* = 300, PSG-diagnosed), conducting preliminary analyses of seven indices: MHR, PHR, NHHR, AIP, UHR, RC/HDL, and SIRI. Phase II employed the US National Health and Nutrition Examination Survey (NHANES) database (*n* = 4,423, questionnaire-diagnosed) for external validation, incorporating the CMI index. Multivariate logistic regression models analyzed marker-OSA associations, with area under the ROC curve (AUC) assessing discriminatory capacity and subgroup analyses conducted.

**Results:**

After adjusting for demographics, lifestyle factors, and clinical comorbidities, multiple indicators were independently associated with OSA risk. Within the clinical cohort, AIP, UHR, and RC/HDL demonstrated the most robust associations; in the NHANES cohort, CMI (AUC = 0.621), UHR (AUC = 0.613), AIP, and RC/HDL (both AUC = 0.602) exhibited favorable predictive performance. Subgroup analyses revealed that the predictive value of these markers was particularly pronounced in individuals aged≤60 years, females, non-obese individuals, and those without underlying conditions (hypertension, diabetes, cardiovascular disease).

**Conclusion:**

This two-phase study identified several readily available inflammatory-metabolic composite indices (e.g., MHR, AIP, CMI) as independently associated with OSA risk; these markers demonstrate potential as adjunctive tools for assessing OSA risk. Their predictive efficacy exhibits population heterogeneity, necessitating consideration of individual characteristics in clinical application. Prospective studies are required to further validate their causal associations and clinical utility.

## 1 Background

Obstructive Sleep Apnea (OSA) is a globally prevalent sleep-disordered breathing characterized by recurrent upper airway collapse and intermittent hypoxia (IH) during sleep (1, 2). It is estimated that the prevalence of OSA in the global adult population ranges from 9 to 38%, affecting approximately one billion individuals. Its incidence continues to rise alongside the prevalence of obesity, presenting a significant public health challenge (3–5). Research confirms that OSA patients, regardless of the presence of typical clinical symptoms, exhibit a markedly increased risk of hypertension, cardiovascular disease, and stroke (1, 5, 6). Moreover, the chronic hypoxia associated with OSA can lead to excessive daytime sleepiness, impaired concentration, memory deficits, and cognitive decline. This not only reduces work efficiency and increases error rates but also substantially elevates the risk of occupational accidents and traffic incidents, resulting in personal injury, property damage, and exacerbating socioeconomic and healthcare burdens (7–9). Therefore, timely diagnosis and effective management of OSA are crucial for preventing and controlling its multifaceted health hazards. However, the current gold standard for clinical diagnosis, polysomnography (PSG), typically requires overnight hospitalization. This approach presents limitations including high costs, lengthy waiting times, and poor accessibility, creating diagnostic barriers for many patients (2, 7). Consequently, the early identification, timely diagnosis, and effective management of OSA have become urgent priorities for improving patient health outcomes and alleviating public health pressures.

OSA primarily manifests as intermittent hypoxia and fragmented sleep. Through pathways such as activating the sympathetic nervous system, inducing inflammatory responses, and causing oxidative stress, it leads to metabolic syndrome symptoms characterized by insulin resistance, hypertension, and dyslipidaemia in patients (1, 10–12). Given the strong association between OSA and dyslipidaemia, chronic inflammation, and metabolic dysfunction, hematological biomarkers have been the subject of considerable attention and exploration, with the aim of identifying simple indicators that reflect the pathological state of OSA or predict its risk. Established studies demonstrate that several recently developed, more practical measurement indices—including the non-high-density lipoprotein cholesterol to high-density lipoprotein cholesterol ratio (NHHR), plasma atherogenicity index (AIP), and cardiometabolic index (CMI)—serve as reliable indicators directly pointing to atherosclerosis (13–15). These indices typically integrate information from distinct pathophysiological pathways associated with OSA, potentially offering a more comprehensive assessment than individual reference data.

Therefore, this study first conducted a preliminary assessment of the association between seven novel composite indicators—MHR, PHR, NHHR, AIP, UHR, RC/HDL-C, and SIRI—and OSA risk, based on single-centre clinical data from Chengdu Third People’s Hospital. Subsequently, to validate the generalisability of these findings and broaden the scope of evaluation, we conducted external validation using the nationally representative US National Health and Nutrition Examination Survey (NHANES) database, incorporating the CMI indicator into the analysis at this stage. This research pathway, progressing from clinical cohort exploration to large-scale population validation, aims to systematically identify stable hematological biomarkers closely associated with OSA. This will provide scientific evidence for developing simpler, more cost-effective OSA screening and auxiliary diagnostic tools, thereby helping to overcome the current limitations of PSG in terms of accessibility and convenience, and promoting the early detection and management of OSA.

## 2 Method

### 2.1 Study population

**Figure 1:**
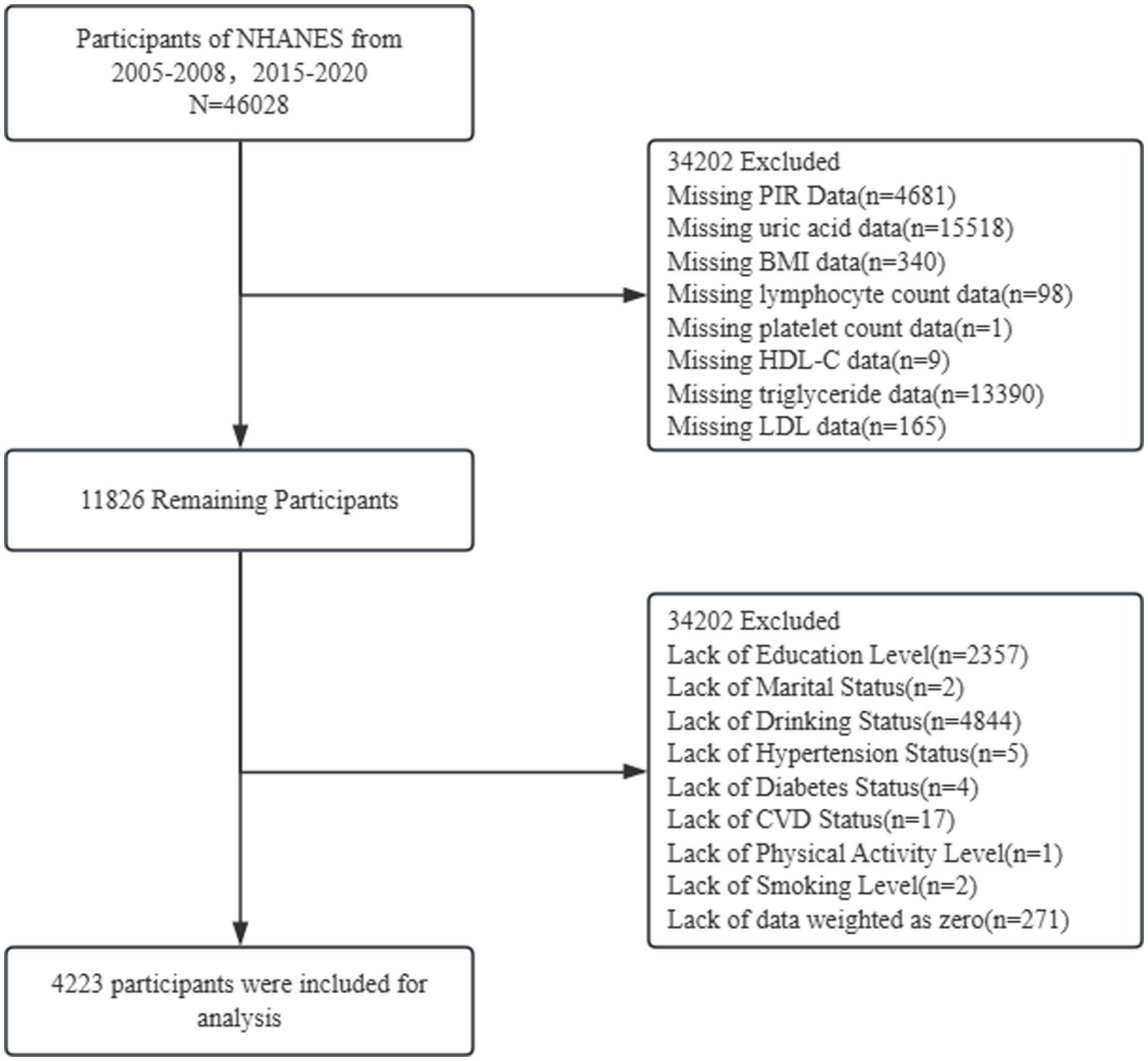
Flowchart of subject selection. The selection of eligible participants from the National Health and Nutrition Examination Survey 2005–2008, 2015–2020.

This study employed a cross-sectional design utilizing clinical cohort data from Chengdu Third People’s Hospital in China alongside publicly available data from the US National Health and Nutrition Examination Survey (NHANES). Clinical cohort data: Information was collected on OSA patients attending the Sleep Medicine Centre at Chengdu Third People’s Hospital between 2023 and 2025. NHANES data: Data from four cycles (2005–2006, 2007–2008, 2015–2016, 2017–2020) were extracted, encompassing demographic, lifestyle, clinical examination, and laboratory testing information for external validation (see Figure 1).

### 2.2 Definition of obstructive sleep apnea

Clinical Cohort: Patients diagnosed with OSA based on polysomnography (PSG) results (AHI ≥ 5) were enrolled as the case group. The control group comprised individuals with simple snoring who visited the Sleep Medicine Center during the same period and were confirmed to have no OSA by PSG (AHI < 5). Due to the relatively small sample size of the clinical cohort, we conducted matching in a 1:1 ratio to select the control group.

NHANES: According to earlier studies, the presence of OSA-related symptoms can be diagnosed if the answer to at least one of the following three questions from NHANES is “yes” (16, 17): (1) Excessive daytime sleepiness despite sleeping at least 7 h per night, reporting 16 to 30 episodes; (2) Episodes of gasping, snorting, or breathing cessation occurring three or more times weekly; (3) Snoring three or more times weekly.

### 2.3 Laboratory tests: blood cell count, cholesterol

**Table:** 

Chengdu Third People’s Hospital Laboratory Measurements	
Triglycerides	Enzymatic method: GPO-POD
Total cholesterol	Cholesterol oxidase method
HDL	Selective inhibition method (PPD method)
LDL	Surfactant removal method (SUR method)
Complete blood count and five-part differential	Fluorescent staining flow cytometry

NHANES performed complete blood cell counts on blood samples using the Beckman Coulter MAXM instrument in the laboratory, providing blood cell distribution for all participants. For subjects undergoing fasting examinations, triglyceride and cholesterol levels were analysed using the Roche Modular P chemistry analyser. LDL cholesterol was calculated using the Friedewald formula based on measured values of total cholesterol, triglycerides, and HDL cholesterol.

Calculate the following eight indices using the aforementioned test data:

**Table:** 

Indicator	Calculation method
MHR	Monocyte Count / HDL-C
PHR	Platelet Count / HDL-C
NHHR	Non-HDL Cholesterol / HDL-C
AIP	log(Triglycerides / HDL-C)
UHR	Uric Acid / HDL-C
RC/HDL	Residual cholesterol / HDL-C
SIRI	Neutrophil count × Monocyte count / Lymphocyte count
CMI	Triglycerides × Waist circumference / HDL-C

### 2.4 Covariates

Clinical Cohort: Diabetes Diagnosis: Diabetes was diagnosed following a 72-gram oral glucose tolerance test when fasting blood glucose ≥7.0 mmol/L, or glycated hemoglobin ≥6.5%, or random blood glucose ≥11.1 mmol/L, or 2-h blood glucose ≥11.1 mmol/L. Hypertension was diagnosed based on three or more blood pressure measurements taken on different dates, with systolic pressure ≥140 mmHg and/or diastolic pressure ≥90 mmHg. Smoking and alcohol consumption history: Patients self-reported their overall smoking/drinking status.

Based on existing NHANES literature and biological plausibility, this study incorporated the following variables as covariates: age, gender, race/ethnicity, marital status, smoking status (18), drinking status (19), educational attainment, hypertension (20), diabetes (21), and the poverty income ratio (PIR).

Furthermore, cardiovascular events were defined as a self-reported history of cardiovascular disease (CVD), where participants answered “yes” to any of the following questions: “Has a doctor or other health professional ever told you that you have heart failure, coronary heart disease, myocardial infarction, angina pectoris, or stroke?.” The outcome measure was a composite endpoint of any of the aforementioned cardiovascular events (including heart failure, coronary heart disease, myocardial infarction, angina pectoris, or stroke) (22). Physical activity was assessed based on whether participants engaged in walking or cycling and was included as a covariate in the analysis (23).

### 2.5 Statistical methods

In the clinical cohort analysis, we employed logistic regression models to investigate relevant risk factors. Model I was unadjusted for covariates; Model II adjusted for age (continuous variable), sex, smoking status, and alcohol consumption; Model III further incorporated BMI, hypertension, and history of diabetes mellitus on top of Model II.

In accordance with NHANES recommendations, this analysis employed NHANES MEC examination weights to ensure national representativeness. Baseline characteristics of the study population are presented as weighted means ± standard deviation for continuous variables and as unweighted case counts and weighted percentages for categorical variables. In the logistic regression models for obstructive sleep apnea (OSA), we accounted for the effects of the complex survey design and employed a stepwise adjustment approach to assess potential confounding effects of covariates. Model I was the unadjusted model; Model II adjusted for age, sex, ethnicity, educational attainment, marital status, and household income ratio (PIR); Model III further incorporated alcohol consumption status, BMI, hypertension, history of diabetes, history of cardiovascular disease, physical activity level, and smoking status on top of Model II.

To investigate potential heterogeneity, we conducted subgroup analyses by sex (male/female), age, BMI (≤25, 25–30, >30 kg/m^2^), race/ethnicity, marital status, smoking status, drinking status, educational attainment, PIR, physical activity, and presence of diabetes or hypertension, as well as occurrence of cardiovascular events. Additionally, a restricted cubic spline (RCS) analysis was employed to examine the dose–response relationship between the eight indicators and OSA occurrence. All statistical analyses utilized two-tailed tests, with a significance level set at *p* < 0.05. To assess the stability of the predictive performance of biomarkers in both the clinical cohort and the NHANES cohort, we used the DeLong method to calculate the 95% confidence intervals for the AUC of each inflammatory-metabolic index. Additionally, 10-fold cross-validation was conducted to obtain bias-corrected AUC estimates. For the NHANES cohort study, unweighted logistic regression was used for sensitivity analysis to assess the reliability of the association between inflammatory-metabolic indicators and obstructive sleep apnea symptoms.

## 3 Result

### 3.1 Clinical data analysis

#### 3.1.1 Clinical data study population

This study employed a cross-sectional design, with data sourced from clinical records at Chengdu Third People’s Hospital between 2023 and 2025. A total of 300 participants were enrolled. Based on polysomnography (PSG) results, 150 patients with an apnea-hypopnea index (AHI) ≥ 5 events per hour constituted the obstructive sleep apnea (OSA) group, while 150 subjects with AHI < 5 events per hour during the same period formed the control group. Body mass index (BMI), gender, history of hypertension, history of diabetes, smoking history, and alcohol consumption history were collected from all participants as covariates.

Table 1 presents the baseline characteristics of the study subjects. Compared with the control group, the OSA group exhibited statistically significant differences in most baseline indicators (all *p* values<0.05 except for gender). Within the OSA group, overweight individuals constituted the highest proportion (44.7%), followed by those with normal BMI (30.0%) and obese individuals (25.3%). Furthermore, the prevalence of hypertension was significantly higher in the OSA group than in the control group. Regarding serological indicators, levels of MHR, PHR, NHHR, UHR, and RC/HDL were all significantly elevated in the OSA group compared to the control group.

**Table 1:** Characteristics of Chengdu Third People's Hospital Study participants classified by obstructive sleep apnea from 2023 to 2025.

Characteristics	Overall (*N* = 300)	Without OSA (*N* = 150)	With OSA (*N* = 150)	*P* value
Gender, *n*, %				0.106
Male	143 (47.7)	64 (42.7)	79 (52.7)	
Female	157 (52.3)	86 (57.3)	71 (47.3)	
Age(years), mean ± SD	44.60 ± 13.99	50.99 ± 13.73	38.21 ± 11.04	<0.001
Smoking status, *n*, %				0.015
Yes	105 (35.0)	42 (28.0)	63 (42.0)	
No	195 (65.0)	108 (72.0)	87 (58.0)	
Drinking status, *n*, %				<0.001
Yes	105 (35.0)	35 (23.3)	70 (46.7)	
No	195 (65.0)	115 (76.7)	80 (53.3)	
BMI, *n*, %				<0.001
< = 25	143 (47.7)	98 (65.3)	45 (30.0)	
25–30	112 (37.3)	45 (30.0)	67 (44.7)	
>30	45 (15.0)	7 (4.7)	38 (25.3)	
Hypertension, *n*, %				0.016
Yes	88 (29.3)	34 (22.7)	54 (36.0)	
No	212 (70.7)	116 (77.3)	96 (64.0)	
Diabetes, *n*, %				0.031
Yes	35 (11.7)	24 (16.0)	11 (7.3)	
No	265 (88.3)	126 (84.0)	139 (92.7)	
MHR, mean ± SD	0.29 ± 0.15	0.25 ± 0.12	0.33 ± 0.16	<0.001
PHR, mean ± SD	182.38 ± 74.67	152.56 ± 60.87	212.21 ± 75.44	<0.001
NHHR, mean ± SD	2.95 ± 1.03	2.68 ± 0.97	3.22±1.03	<0.001
AIP, mean ± SD	0.09 ± 0.32	−0.11 ± 0.30	−0.20 ± 0.31	<0.001
UHR, mean ± SD	0.30 ± 0.13	0.24 ± 0.10	0.35 ± 0.13	<0.001
RC/HDL, mean ± SD	0.55 ± 0.42	0.46 ± 0.29	0.63 ± 0.50	<0.001
SIRI, mean ± SD	0.89 ± 0.71	0.91 ± 0.76	0.88 ± 0.66	0.746

OSA, obstructive sleep apnea; BMI, body mass index; PIR: family income-poverty ratio; MHR, Monocyte to HDL Ratio; PHR, Platelet to HDL Ratio; NHHR, Non-HDL to HDL Ratio; AIP, atherogenic Index of Plasma; UHR, Uric acid to HDL Ratio; RC/HDL, Remnant Cholesterol to HDL Ratio; SIRI, Systemic Inflammation Response Index.

#### 3.1.2 Association between multiple serum composite indicators and OSA

To assess the independent association between levels of multiple serum composite markers and obstructive sleep apnea (OSA), researchers conducted weighted multivariate logistic regression analyses, with results presented in Table 2.

**Table 2:** Multivariable logistic regression analysis of multiple serum composite indicators and obstructive sleep apnea.

Variables	Model I OR (95% CI)	*P*-value	Model II OR (95% CI)	*P*-value	Model III OR (95% CI)	*P*-value
MHR						
Q1 0.08–0.20	Ref		Ref		Ref	
Q2 0.20–0.26	2.92 (1.46, 5.87)	0.003	2.61 (1.18, 5.79)	0.018	1.80 (0.76, 4.31)	0.184
Q3 0.26–0.35	4.75 (2.35, 9.59)	<0.001	3.90 (1.72, 8.85)	0.001	3.10 (1.23, 7.79)	0.016
Q4 0.35–1.22	6.73 (3.28, 13.80)	<0.001	4.40 (1.87, 10.32)	<0.001	2.26 (0.85, 6.01)	0.101
PHR						
Q1 40.98–126.49	Ref		Ref		Ref	
Q2 126.49–174.01	2.11 (1.05, 4.26)	0.037	1.45 (0.66, 3.19)	0.349	1.08 (0.45, 2.58)	0.861
Q3 174.01–222.12	4.49 (2.23, 9.06)	<0.001	2.47 (1.09, 5.62)	0.031	1.59 (0.63, 4.00)	0.327
Q4 222.12-476.71	10.80(5.07,23.03)	<0.001	5.62(2.38,13.32)	<0.001	3.20 (1.21, 8.49)	0.019
NHHR						
Q1 0.54–2.20	Ref		Ref		Ref	
Q2 2.20–2.91	2.82 (1.43, 5.59)	0.003	2.01 (0.92, 4.35)	0.078	1.59 (0.67, 3.73)	0.290
Q3 2.91–3.63	3.70 (1.86, 7.34)	<0.001	2.74 (1.24, 6.02)	0.013	1.64 (1.68, 3.95)	0.267
Q4 3.63–9.87	5.18 (2.58, 10.42)	<0.001	3.41 (1.52, 7.64)	0.003	2.00 (0.81, 4.93)	0.135
AIP						
Q1 (−0.68)–(−0.13)	Ref		Ref		Ref	
Q2 (−0.13)–0.08	2.92 (1.46, 5.87)	0.003	3.33 (1.50, 7.39)	0.003	2.43 (1.02, 5.74)	0.044
Q3 0.08–0.29	5.02 (2.48, 10.16)	<0.001	4.85 (2.16, 10.87)	<0.001	2.49 (1.00, 6.21)	0.050
Q4 0.29–1.28	6.33 (2.10, 12.95)	<0.001	5.35 (2.32, 12.31)	<0.001	2.90 (1.12, 7.53)	0.028
UHR						
Q1 0.00–0.19	Ref		Ref		Ref	
Q2 0.19–0.28	2.38 (1.14, 4.97)	0.020	2.54 (1.11, 5.82)	0.027	1.60 (0.66, 3.87)	0.301
Q3 0.28–0.38	6.71 (3.22, 13.99)	<0.001	5.59 (2.35, 13.32)	<0.001	2.94 (1.13, 7.65)	0.027
Q4 0.38–0.90	16.00 (7.19, 35.61)	<0.001	10.0 8(3.98, 25.55)	<0.001	4.33 (1.52, 12.37)	0.006
RC/HDL						
Q1 (−1.00)–0.34	Ref		Ref		Ref	
Q2 0.34–0.48	3.19 (1.60, 6.37)	<0.001	3.27 (1.47, 7.26)	0.004	2.82 (1.18, 6.75)	0.020
Q3 0.48–0.68	5.24 (2.60, 10.57)	<0.001	5.07 (2.23, 11.54)	<0.001	3.36 (1.38, 8.16)	0.007
Q4 0.68–5.51	4.18 (2.09, 8.38)	<0.001	3.83 (1.68, 8.74)	0.001	2.52 (1.03, 6.14)	0.042
SIRI						
Q1 0.15–0.49	Ref		Ref		Ref	
Q2 0.49–0.72	0.71 (0.37, 1.35)	0.292	0.56 (0.26, 1.19)	0.130	0.65 (0.28, 1.54)	0.329
Q3 0.72–1.07	1.28 (0.67, 2.43)	0.458	1.10 (0.52, 2.35)	0.806	1.06 (0.46, 2.47)	0.886
Q4 1.07–7.71	1.24 (0.65, 2.35)	0.513	1.11 (0.52, 2.35)	0.786	1.11 (0.47, 2.60)	0.809

Model I: unadjusted model. Model II: adjusted for age, gender, drinking status, smoking status. Model III: adjusted for variables in Model II plus BMI, hypertension, diabetes status. BMI, body mass index; MHR, Monocyte to HDL Ratio; PHR, Platelet to HDL Ratio; NHHR, Non-HDL to HDL Ratio; AIP, Atherogenic Index of Plasma; UHR, Uric acid to HDL Ratio; RC/HDL, Remnant Cholesterol to HDL Ratio; SIRI, Systemic Inflammation Response Index; OR, odds ratio; 95% CI, 95% confidence interval.

In the unadjusted Model I, higher quartiles (Q2, Q3, and Q4) of MHR, PHR, NHHR, AIP, UHR, and RC/HDL predominantly showed significant positive correlations with OSA risk. After adjusting for age, sex, alcohol consumption, and smoking status (Model II), most of these positive associations remained statistically significant. Upon further adjustment for BMI, hypertension, and diabetes status (Model III), the strength of some associations diminished; however, multiple indicators including MHR, PHR, AIP, UHR, and RC/HDL were still observed to be independently associated with OSA risk.

**Figure 2:**
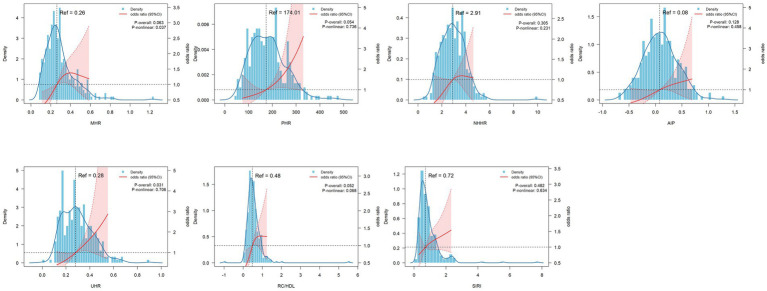
RCS analysis of multiple inflammatory metabolic composite index levels with obstructive sleep apnea in clinical cohort. Adjusted for age, gender, BMI, smoking status, drinking status, hypertension, diabetes. OSA, obstructive sleep apnea; BMI, body mass index; MHR, Monocyte to HDL ratio; PHR, Platelet to HDL ratio; NHHR, Non-HDL to HDL ratio; AIP, atherogenic index of plasma; UHR, Uric acid to HDL ratio; RC/HDL, Remnant cholesterol to HDL ratio; SIRI, Systemic inflammation response index.

Specifically, in fully adjusted Model III, the Q3 quartile of MHR was significantly associated with OSA risk compared to the Q1 quartile (OR = 3.10, 95% CI = 1.23–7.79, *p* = 0.016), whereas no significant association was observed for the Q2 and Q4 quartiles; For PHR, only the Q4 group showed a significant positive correlation (OR = 3.20, 95% CI = 1.21–8.49, *p* = 0.019); none of the NHHR quantiles demonstrated significant associations in Model III; all AIP quantiles (Q2: OR = 2.43, *p* = 0.044; Q3: OR = 2.49, *p* = 0.050; and Q4: OR = 2.90, *p* = 0.028) were significantly associated with OSA risk; Q3 and Q4 UHR groups (OR = 2.94, *p* = 0.027 and OR = 4.33, *p* = 0.006, respectively) exhibited significantly elevated risk; Q2 to Q4 groups of RC/HDL showed significant positive correlations (OR = 2.82, *p* = 0.020; OR = 3.36, *p* = 0.007; OR = 2.52, *p* = 0.042 respectively); whereas SIRI demonstrated no significant association across all quantiles in Model III (see Figure 2).

#### 3.1.3 ROC curve analysis

Within the overall cohort (n = 300), all serum composite indicators demonstrated excellent discriminatory capability, with Area Under the Curve (AUC) values significantly exceeding 0.5 and ranging between 0.867 and 0.875. Among these, PHR and UHR both achieved an AUC of 0.875, representing the optimal performance; SIRI exhibited the relatively lowest AUC of 0.867, though this difference was not statistically significant.

Subgroup analysis revealed particularly pronounced discriminatory efficacy among individuals aged >60 years. NHHR achieved an AUC approaching 1.000, with PHR at 0.959 and UHR at 0.955. In the ≤60 age cohort, AUC values were slightly lower than in the older group, yet discriminatory performance remained highly effective, with UHR (AUC = 0.814) and PHR (AUC = 0.806) demonstrating favorable outcomes.

After stratification by gender, AUC values were higher across all indicators in the female cohort, with UHR exhibiting the highest AUC (AUC = 0.912); in the male cohort, AUC values were slightly lower, with PHR demonstrating the highest AUC (AUC = 0.883).

**Figure 3:**
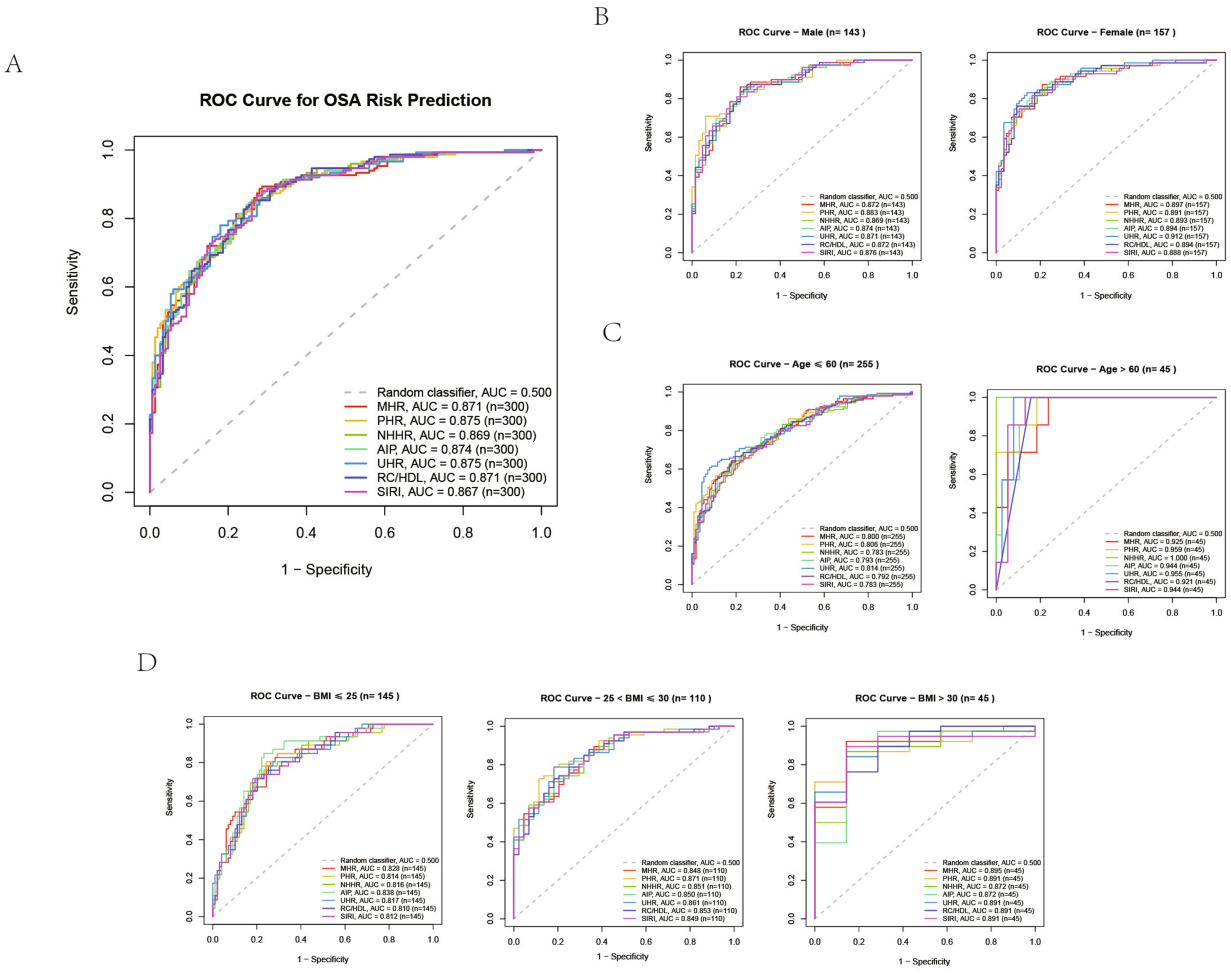
ROC curves for the predictive performance of the Multiple Inflammatory Metabolic Composite Index for OSA in clinical cohort. **(A)** Total population. **(B)** Gender-stratified subgroups. **(C)** Age-stratified subgroups. **(D)** BMI-stratified subgroups.

Across different BMI groups, all indicators maintained comparable high discriminatory capabilities. The AUC range for the normal BMI group was 0.810–0.838, with AIP yielding the highest value (AUC = 0.838); for the overweight group, the range was 0.848–0.871, with PHR achieving the highest AUC (AUC = 0.871); for the obese group, the range was 0.872–0.895, with MHR demonstrating the highest AUC (AUC = 0.895) (see Figure 3).

To assess the stability of the AUC estimates in the clinical cohort, we conducted an internal validation using the DeLong method and obtained the 95% confidence interval of the AUC. The optimal cutoff values, sensitivity, specificity, and confidence intervals of the AUC for all markers can be found in Supplementary Table 1.

### 3.2 Analysis of the NHANES database

#### 3.2.1 Study population

Our analysis examined data from four NHANES cycles spanning 2005–2006, 2007–2008, 2015–2016, and 2017–2020, encompassing 46,028 participants. After excluding individuals lacking blood sample measurements and anthropometric data, as well as those with missing information on educational attainment, marital status, alcohol consumption, smoking habits, hypertension status, cardiovascular disease status, and diabetes status, and excluding cases with a sample weight of zero, the final analysis sample comprised 4,423 participants. Among these, 1,902 individuals were diagnosed with OSA.

Table 3 presents baseline characteristics of study participants stratified by OSA status. Among the 4,423 subjects ultimately included, the weighted prevalence of OSA was 43.00% (*n* = 1,902). Compared with those without OSA, individuals with OSA exhibited significant differences in multiple baseline characteristics (all *p* values <0.05 except for ethnicity, alcohol consumption history, daily activities, and CVDs). The OSA cohort exhibited a higher mean age, a slightly greater proportion of females, and predominantly high school or higher educational attainment. Socioeconomically, a higher proportion were married or cohabiting (70.6%), though fewer lived in poverty (PIR ≤ 1.3); non-smokers constituted a larger proportion (55.4%). Clinically, BMI distribution in the OSA group showed a positive correlation, with the highest proportion being obese (53.4%), followed by overweight (30.3%), and then normal BMI (16.3%). Prevalence of hypertension and diabetes was markedly higher than in the non-OSA group. Furthermore, their MHR, PHR, NHHR, AIP, UHR, RC/HDL, SIRI, and CMI were all higher.

**Table 3:** Characteristics of NHANES study participants classified by obstructive sleep apnea from 2005 to 2008 and from 2015 to 2020.

Characteristics	Overall (*N* = 4223)	Without OSA (*N* = 2321)	With OSA (*N* = 1902)	*P* value
Gender, *n*, %				<0.001
Male	1,790 (43.1)	876 (38.4)	914 (49.5)	
Female	2,433 (56.9)	1,445 (61.6)	988 (50.5)	
Age(years), Mean ± SD	48.26 ± 17.13	46.75 ± 18.00	50.31 ± 15.64	<0.001
Race, *n*, %				0.092
Mexican American	655 (9.0)	333 (8.1)	322 (10.2)	
Non-Hispanic Black	1,032 (12.2)	562 (11.7)	470 (12.8)	
Non-Hispanic White	1,587 (64.1)	920 (66.0)	667 (61.5)	
Other Hispanic	389 (5.8)	194 (5.3)	195 (6.5)	
Other Race	560 (9.0)	312 (8.8)	248 (9.1)	
Education level, *n*, %				0.010
Below high school	904 (12.8)	496 (11.9)	408 (14.0)	
High school grad/GED or equivalent	1,013 (25.8)	539 (23.6)	474 (28.7)	
Above high school	2,306 (61.4)	1,286(64.5)	1,020 (57.3)	
Marital status, *n*, %				<0.001
Married/living with partner	2,572 (63.4)	1,308 (58.2)	1,264 (70.6)	
Never married	708(17.8)	471 (21.9)	237 (12.2)	
Widowed/divorced/separated	943(18.8)	542 (19.9)	401 (17.3)	
PIR, *n*, %				0.021
< = 1.3	1,206 (19.4)	684 (18.5)	522 (20.5)	
1.3–3.5	1,716 (36.5)	915 (34.4)	801 (39.5)	
>3.5	1,301 (44.1)	722 (47.1)	579 (39.9)	
Drinking status, *n*, %				0.393
Yes	2,198 (56.3)	1,157 (55.4)	1,041 (57.6)	
No	2,025 (43.7)	1,164 (44.6)	8,61 (42.4)	
Smoking status, *n*, %				0.001
Yes	1633(39.3)	807(35.3)	826(44.6)	
No	2590(60.7)	1514(64.7)	1076(55.4)	
BMI, *n*, %				<0.001
< = 25	1,104 (27.7)	783 (36.1)	321 (16.3)	
25–30	1,336 (31.6)	757 (32.6)	579 (30.3)	
>30	1,783 (40.7)	781 (31.3)	1,002 (53.4)	
Physical activity level, *n*, %				0.467
Yes	936 (20.4)	532 (20.9)	404 (19.7)	
No	3,287 (79.6)	1,789 (79.1)	1,498 (80.3)	
Hypertension, *n*, %				<0.001
Yes	1,611 (32.8)	785 (28.5)	826 (38.5)	
No	2,612 (67.2)	1,536 (71.5)	1,076 (61.5)	
Diabetes, *n*, %				<0.001
Yes	739 (12.4)	321 (9.5)	418 (16.4)	
No	3,484 (87.6)	2,000 (90.5)	1,484 (83.6)	
CVD, n, %				0.888
Yes	495 (9.4)	250 (9.5)	245 (9.3)	
No	3,728 (90.6)	2,071 (90.5)	1,657 (90.7)	
MHR, mean ± SD	0.42 ± 0.21	0.40 ± 0.21	0.46 ± 0.21	<0.001
PHR, mean ± SD	190.60 ± 74.58	183.07 ± 72.34	200.83 ± 76.37	<0.001
NHHR, mean ± SD	2.66 ± 1.14	2.52 ± 1.11	2.85 ± 1.16	<0.001
AIP, mean ± SD	−0.11 ± 0.32	−0.15 ± 0.32	−0.04 ± 0.30	<0.001
UHR, mean ± SD	0.11 ± 0.05	0.10 ± 0.04	0.12 ± 0.05	<0.001
RC/HDL, mean ± SD	0.47 ± 0.37	0.42 ± 0.34	0.53 ± 0.39	<0.001
SIRI, mean ± SD	1.18 ± 0.77	1.15 ± 0.78	1.22 ± 0.76	0.006
CMI, mean ± SD	0.64 ± 0.54	0.56 ± 0.50	0.74 ± 0.58	<0.001

OSA, obstructive sleep apnea; BMI, body mass index; PIR: family income-poverty ratio; MHR, Monocyte to HDL Ratio; PHR, Platelet to HDL Ratio; NHHR, Non-HDL to HDL Ratio; AIP, atherogenic Index of Plasma; UHR, Uric acid to HDL Ratio; RC/HDL, Remnant Cholesterol to HDL Ratio; SIRI, Systemic Inflammation Response Index; CMI, Cardiometabolic Index.

#### 3.2.2 Association between multiple serum composite indicators and OSA

To assess the independent association between levels of multiple serum composite indicators and obstructive sleep apnea (OSA), researchers conducted weighted multivariate logistic regression analysis, with results presented in Table 4.

**Table 4:** Weighted multivariable logistic regression analysis of multiple serum composite indicators and obstructive sleep apnea.

	Model I OR (95% CI)	*P*-value	Model II OR (95% CI)	*P*-value	Model III OR (95% CI)	*P*-value
MHR						
Q1 0.04–0.28	Ref		Ref		Ref	
Q2 0.28–0.38	1.51 (1.20, 1.90)	0.001	1.49 (1.19, 1.88)	0.001	1.24 (0.99, 1.57)	0.068
Q3 0.38–0.52	1.54 (1.24, 1.92)	<0.001	1.46 (1.17, 1.81)	0.001	1.06 (0.84, 1.34)	0.636
Q4 0.52–3.67	2.52 (2.05, 3.09)	<0.001	2.23 (1.79, 2.77)	<0.001	1.48 (1.18, 1.87)	0.001
PHR						
Q1 30.06–135.82	Ref		Ref		Ref	
Q2 135.82–179.28	1.33 (0.96, 1.84)	0.095	1.36 (0.98, 1.87)	0.067	1.16 (0.83,1.62)	0.379
Q3 179.28–231.67	1.70 (1.30, 2.23)	<0.001	1.75 (1.35, 2.28)	<0.001	1.31 (1.00, 1.72)	0.057
Q4 231.67–817.33	2.08 (1.66, 2.62)	<0.001	2.17 (1.70, 2.75)	<0.001	1.46 (1.14, 1.87)	0.004
NHHR						
Q1 0.28–1.80	Ref		Ref		Ref	
Q2 1.80–2.47	1.93 (1.47, 2.54)	<0.001	1.88 (1.41, 2.49)	<0.001	1.55 (1.51, 2.09)	0.006
Q3 2.47–3.31	2.15 (1.71, 2.69)	<0.001	1.97 (1.59, 2.44)	<0.001	1.53 (1.20, 1.96)	0.001
Q4 3.31–9.26	2.41 (1.90, 3.05)	<0.001	2.08 (1.63, 2.65)	<0.001	1.41 (1.08, 1.84)	0.015
AIP						
Q1 (−1.25)–(−0.32)	Ref		Ref		Ref	
Q2 (−0.32)–(−0.11)	1.87 (1.44, 2.43)	<0.001	1.73 (1.34, 2.24)	<0.001	1.44 (1.09, 1.89)	0.013
Q3 (−0.11)-0.13	2.40 (1.85, 3.10)	<0.001	2.09 (1.63, 2.67)	<0.001	1.49 (1.15, 1.94)	0.005
Q4 0.13–0.86	2.62 (2.09, 3.29)	<0.001	2.22 (1.72, 2.86)	<0.001	1.42 (1.07, 1.90)	0.021
UHR						
Q1 0.01–0.07	Ref		Ref		Ref	
Q2 0.07–0.10	1.71 (1.33, 2.20)	<0.001	1.61 (1.26, 2.04)	<0.001	1.18 (0.92, 1.52)	0.204
Q3 0.10–0.14	1.84 (1.37,2.46)	<0.001	1.69 (1.26, 2.26)	0.001	1.09 (0.81, 1.48)	0.563
Q4 0.14–0.43	2.96 (2.30, 3.82)	<0.001	2.61 (2.09, 3.26)	<0.001	1.40 (1.13, 1.75)	0.004
RC/HDL						
Q1 0.03–0.22	Ref		Ref		Ref	
Q2 0.22–0.36	1.89 (1.48, 2.42)	<0.001	1.75 (1.36, 2.25)	<0.001	1.47 (1.13, 1.91)	0.006
Q3 0.36–0.62	2.49 (1.92, 3.22)	<0.001	2.18 (1.70, 2.79)	<0.001	1.55 (1.18, 2.03)	0.003
Q4 0.62–3.29	2.67 (2.12, 3.36)	<0.001	2.26 (1.75, 2.92)	<0.001	1.46 (1.09, 1.96)	0.014
SIRI						
Q1 0.08–0.64	Ref		Ref		Ref	
Q2 0.64–0.97	1.33 (1.05, 1.68)	0.019	1.33 (1.06, 1.66)	0.015	1.24 (0.99, 1.54)	0.065
Q3 0.97–1.46	1.27 (0.97, 1.66)	0.089	1.23 (0.94, 1.63)	0.116	1.01 (0.77, 1.32)	0.942
Q4 1.46–11.60	1.72 (1.38, 2.13)	<0.001	1.55 (1.22, 1.97)	0.001	1.27 (1.01, 1.60)	0.044
CMI						
Q1 0.03–0.27	Ref		Ref		Ref	
Q2 0.27–0.48	1.78 (1.38, 2.31)	<0.001	1.60 (1.23, 2.09)	0.001	1.26 (0.96, 1.67)	0.108
Q3 0.48–0.86	2.75 (2.13, 3.54)	<0.001	2.34(1.86,2.95)	<0.001	1.52 (1.17, 1.97)	0.003
Q4 0.86–5.22	2.82 (2.26, 3.51)	<0.001	2.40 (1.87, 3.)	<0.001	1.29 (0.96, 1.74)	0.021

Model I: unadjusted model. Model II: adjusted for age, gender, race, education level, Marital, PIR. Model III: adjusted for variables in Model II plus drinking status, BMI, hypertension, diabetes status, CVD, physical activity level, smoking status; BMI, body mass index; PIR: family income-poverty ratio; MHR, Monocyte to HDL Ratio; PHR, Platelet to HDL Ratio; NHHR, Non-HDL to HDL Ratio; AIP, atherogenic Index of Plasma; UHR, Uric acid to HDL Ratio; RC/HDL, Remnant Cholesterol to HDL Ratio; SIRI, Systemic Inflammation Response Index; CMI, Cardiometabolic Index; CVD, cardiovascular disease; OR, odds ratio; 95% CI, 95% confidence interval.

In the unadjusted Model I, the higher quartiles (Q2, Q3, and Q4) of all serum composite indicators predominantly exhibited significant positive correlations with OSA risk. Following adjustment for demographic variables (Model II), most of these positive associations remained statistically significant. Following further adjustment for confounding factors including lifestyle and clinical comorbidities (Model III), the strength of these associations generally diminished. Nevertheless, higher quantiles (primarily Q3 and/or Q4) of multiple indicators—including MHR, NHHR, AIP, and RC/HDL—were still independently associated with OSA risk. Conversely, PHR and UHR demonstrated significant associations only at the Q4 level.

**Figure 4:**
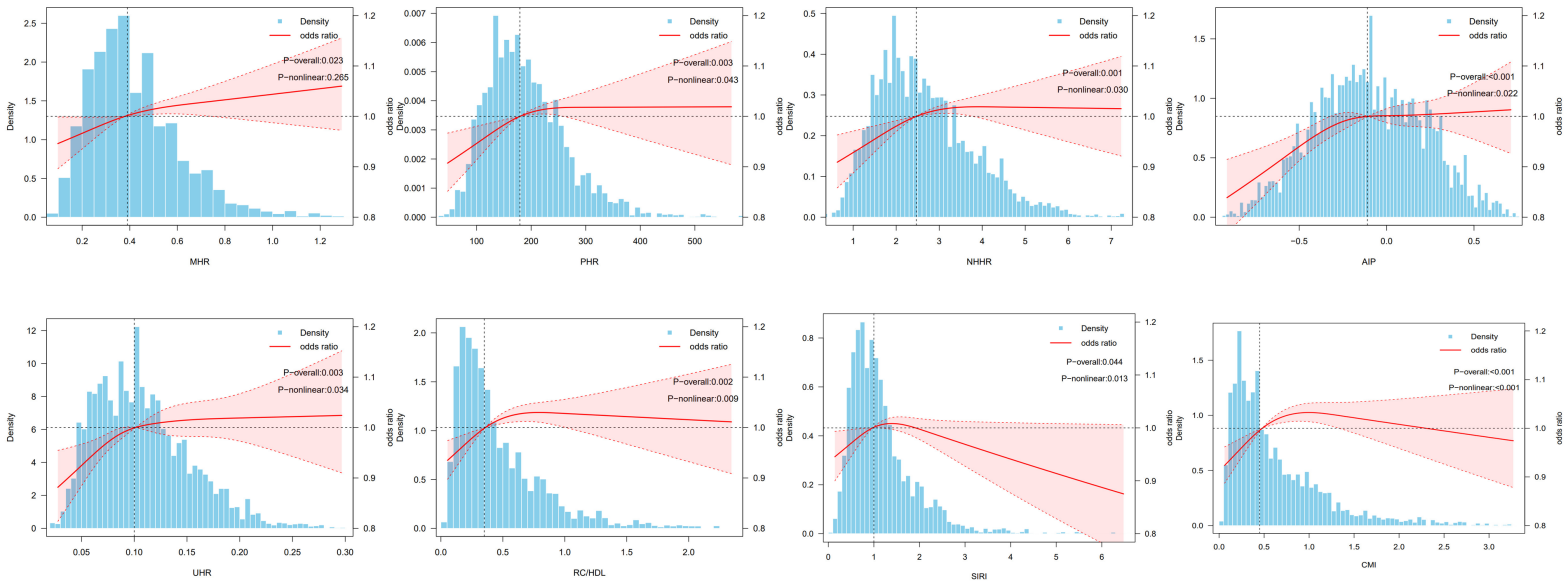
RCS analysis of multiple inflammatory metabolic composite index levels with obstructive sleep apnea in NHANES. Adjusted for age, gender, race, education level, marital status, PIR, drinking status, BMI, hypertension, diabetes, CVD, physical activity level, smoking status. OSA, obstructive sleep apnea; BMI, body mass index; PIR: family income-poverty ratio; CVD, cardiovascular disease; MHR, monocyte to HDL ratio; PHR, platelet to HDL ratio; NHHR, non-HDL to HDL ratio; AIP, atherogenic index of plasma; UHR, uric acid to HDL ratio; RC/HDL, remnant cholesterol to HDL ratio; SIRI, systemic inflammation response index; CMI, cardiometabolic index.

Specifically, in fully adjusted Model III, compared with the first quartile (Q1), the Q4 group of MHR (OR = 1.48, 95% CI = 1.18–1.87, *p* = 0.001), the Q4 group for PHR (OR = 1.46, 95% CI = 1.14–1.87, *p* = 0.004), and the Q2 to Q4 groups for NHHR (OR = 1.55, *p* = 0.006; OR = 1.53, *p* = 0.001; OR = 1.41, *p* = 0.015) all demonstrated significantly elevated OSA risk. The Q2 to Q4 groups for AIP (OR = 1.44, *p* = 0.013; OR = 1.49, *p* = 0.005; OR = 1.42, *p* = 0.021 respectively), the Q2 to Q4 groups for RC/HDL (OR = 1.47, *p* = 0.006; OR = 1.55, *p* = 0.003; OR = 1.46, *p* = 0.014), and the Q4 group of UHR (OR = 1.40, 95% CI = 1.13–1.75, *p* = 0.004) also exhibited significant positive correlations. Furthermore, the SIRI Q4 group (OR = 1.27, 95% CI = 1.01–1.60, *p* = 0.044) and the CMI Q3 group (OR = 1.52, 95% CI = 1.17–1.97, *p* = 0.003) were also significantly associated with OSA risk (see Figure 4).

#### 3.2.3 ROC curve analysis

**Figure 5:**
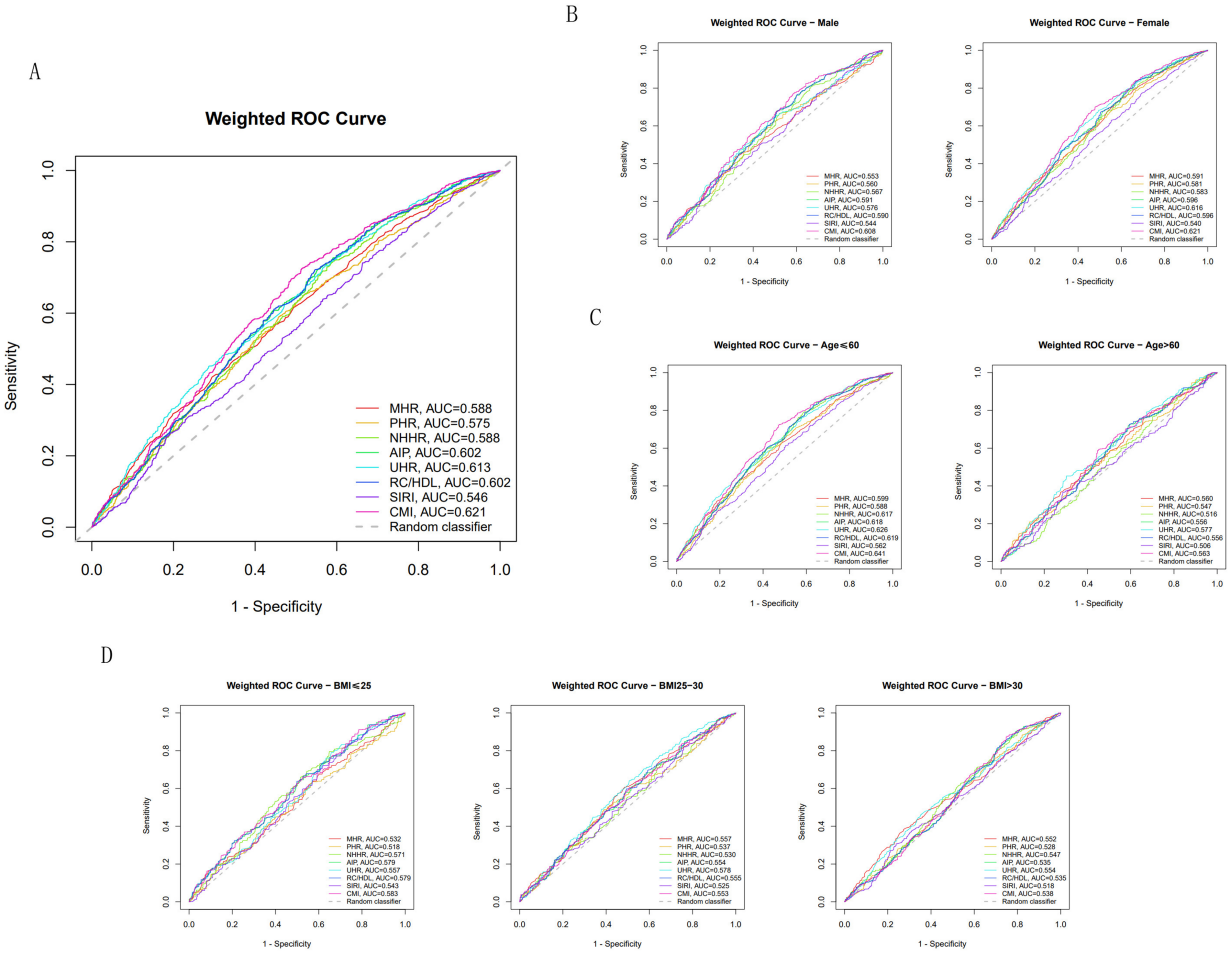
ROC curves for the predictive performance of the multiple inflammatory metabolic composite index for OSA in NHANES. **(A)** Total population. **(B)** Gender-stratified subgroups. **(C)** Age-stratified subgroups. **(D)** BMI-stratified subgroups.

Across the entire cohort, the AUC values for all indicators were significantly above 0.5. Among these, CMI (AUC = 0.621) and UHR (AUC = 0.613) ranked highest, while AIP and RC/HDL (both AUC = 0.602) also demonstrated good and consistent discriminatory power. Traditional MHR and NHHR (AUC 0.588 each) demonstrated moderate performance, while PHR showed slightly weaker performance (AUC = 0.575). SIRI (AUC = 0.546) exhibited the poorest performance among all indicators (see Figure 5).

To further evaluate performance, researchers conducted subgroup analyses stratified by age, sex, and BMI. In the relatively younger cohort aged ≤60 years, all indicators demonstrated superior performance, particularly CMI (AUC = 0.641) and NHHR (AUC = 0.617). In contrast, among the older cohort aged >60 years, AUC values for all indicators showed marked attenuation, with NHHR exhibiting the most pronounced decline (AUC = 0.516). The optimal cutoff values, sensitivity, specificity, and the confidence intervals of AUC for all the markers are all presented in the Supplementary Table 2.

Within the gender-differentiated subgroup, CMI (AUC = 0.621) and UHR (AUC = 0.616) remained the optimal predictive indicators in the female population. Among males, although CMI (AUC = 0.608) maintained its leading position, AIP (AUC = 0.591) and RC/HDL (AUC = 0.590) demonstrated superior predictive value compared to the female cohort.

Furthermore, as BMI categories progressed from normal (≤25) to overweight (25–30) and then to obese (>30), the discriminatory capability of all indicators exhibited a systematic, stepwise decline. Among obese individuals (BMI > 30), the best-performing UHR achieved an AUC of merely 0.554, while SIRI (AUC = 0.518) had virtually lost its discriminatory capacity.

#### 3.2.4 Subgroup analysis

**Figure 6:**
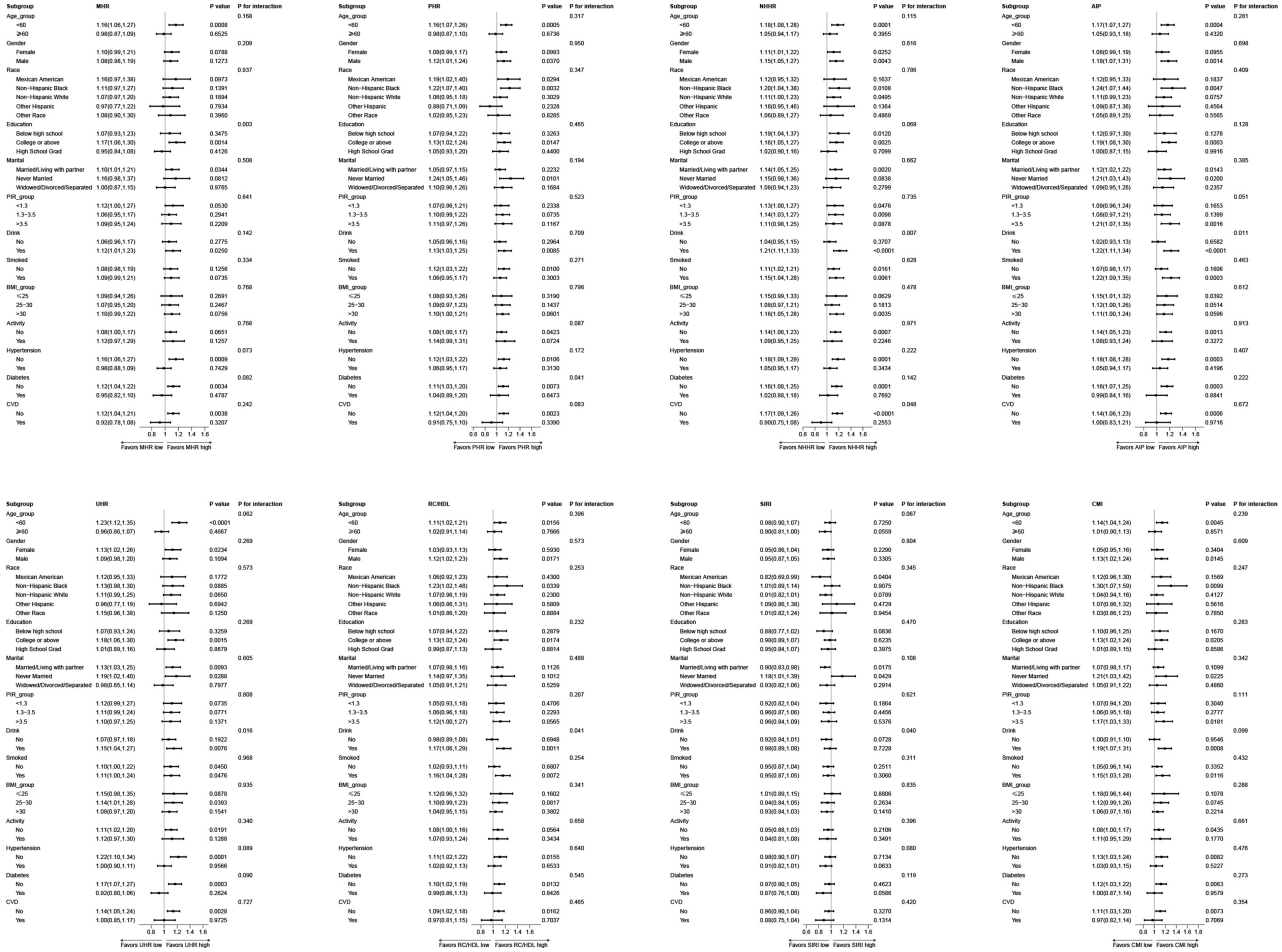
Subgroup and interaction analyses of multiple inflammatory metabolic composite index and OSA from NHANES. Adjusted for age, gender, race, education level, marital, PIR, drinking status, BMI, hypertension, diabetes, CVD, physical activity level, smoking status. All covariates were adjusted for in the subgroup analysis models, except for the stratification variable itself (e.g., “age” was not included as a covariate in the age subgroup analysis). OSA, obstructive sleep apnea; BMI, body mass index; PIR, family income-poverty ratio; CVD, cardiovascular disease; MHR, monocyte to HDL ratio; PHR, platelet to HDL ratio; NHHR, non-HDL to HDL ratio; AIP, atherogenic index of plasma; UHR, uric acid to HDL ratio; RC/HDL, remnant cholesterol to HDL ratio; SIRI, systemic inflammation response index; CMI, cardiometabolic index; 95% CI, 95% confidence interval.

Subgroup analyses revealed that the positive correlation between the standardized eight-item multiple inflammatory metabolic composite score and OSA risk remained broadly consistent across different populations. However, the strength of this association was significantly modified by specific demographic and clinical characteristics. Age emerged as a key modifying factor, with all indicators (except SIRI) demonstrating markedly enhanced risk effects in individuals under 60 years of age (*p* < 0.05 for all indicators except SIRI). Alcohol consumption status also emerged as a critical influencer, with elevated risks for AIP, NHHR, and RC/HDL observed exclusively among drinkers (interaction *p* < 0.05). Furthermore, all markers demonstrated markedly superior predictive value in individuals without underlying conditions (hypertension, diabetes, cardiovascular disease) compared to those with such conditions, particularly NHHR in non-CVD patients (interaction *p* = 0.048). Although interactions between ethnicity did not reach statistical significance, non-Hispanic Blacks exhibited the highest risk ratios across multiple markers including CMI, PHR, and AIP (CMI: OR = 1.30; PHR: OR = 1.22; AIP: OR = 1.24), suggesting potential susceptibility. While marital status had limited overall modifying effects, risk estimates consistently increased across multiple markers among unmarried individuals. Collectively, these findings reveal that age, drinking status, underlying disease status, and racial characteristics are key modifiers influencing the stability of the association between inflammatory metabolic markers and OSA. This provides a basis for their precise application in OSA risk stratification (see Figure 6 for details).

#### 3.2.5 Sensitivity analysis

Consistent with the main research results, the sensitivity analysis further confirmed in the adjusted model that there is a significant positive correlation between these comprehensive indicators and OSA (see Supplementary Table 3). These findings emphasize the stability and consistency of the observed associations between these indicators and OSA under different analytical conditions. However, it is worth noting that the sensitivity analysis results of SIRI have a significant difference from the weighted results, and its robustness may be poor.

## 4 Discussion

This study employed a two-stage analytical strategy, progressing from exploration within a single-centre clinical cohort to validation in a large-scale population database, to systematically evaluate the association between eight inflammatory-metabolic composite indices and the risk of developing obstructive sleep apnea (OSA). Within the clinically diagnosed cohort based on polysomnography (PSG), preliminary analyses revealed significant associations between multiple markers—including MHR, PHR, AIP, UHR, and RC/HDL—and OSA risk. Subsequently, most of these associations were further validated in the nationally representative NHANES cohort, where newly incorporated CMI markers demonstrated superior predictive performance (AUC = 0.621). Notably, AIP and RC/HDL demonstrated stable and independent associations across both heterogeneous cohorts, suggesting robust cross-population applicability as OSA biomarkers. Collectively, the two-stage findings consistently indicate that composite indices integrating multidimensional information—including lipid metabolism and inflammation—possess the robustness required to serve as potential screening tools for OSA.

Our analysis indicates that within clinical cohorts, after adjusting for confounding factors including gender, age, BMI, lifestyle, and clinical comorbidities, the higher quartiles of all serum composite markers—except SIRI and NHHR—exhibit a significant positive correlation with OSA risk. This suggests these inflammatory-metabolic markers may serve as potential biomarkers for OSA risk. ROC curve analysis demonstrated that the AUC for all indicators was significantly greater than 0.50, indicating favorable discriminatory performance. During external validation, we incorporated the novel CMI indicator into the NHANES cohort and found that, after comprehensive adjustment for confounding factors, the higher quartiles of all indicators remained significantly positively correlated with OSA risk, further supporting the aforementioned conclusions. ROC comparisons indicated that CMI and UHR exhibited optimal predictive performance (AUCs of 0.621 and 0.613, respectively), while AIP and RC/HDL also demonstrated good and consistent predictive capability (AUCs of 0.602 each). Subgroup analyses further revealed that the strength of these associations was significantly modulated by factors including age, alcohol consumption status, underlying medical conditions, and ethnicity, providing important evidence for risk stratification and precision screening of OSA.

It is worth noting that there was a significant difference in predictive performance between the clinical cohort and the NHANES cohort in this study, a phenomenon that may be attributed to multiple factors. First, the heterogeneity in the definition of OSA is a key factor: the clinical cohort used polysomnography (PSG) as the gold standard, with cases consisting of OSA patients diagnosed by objective measures and controls comprising simple snorers who were strictly screened for OSA; this design maximized the distinction between cases and controls; In contrast, the NHANES cohort’s questionnaire-based symptom definition has lower specificity, potentially leading to the inclusion of undiagnosed mild OSA patients in the control group and simple snoring in the case group. This non-differential misclassification dilutes the true association strength, causing the AUC to approach 0.5. Second, differences in population characteristics: The clinical cohort consists of hospital-based patients with relatively severe conditions and multiple comorbidities; in contrast, NHANES represents a community-based population that includes a large number of asymptomatic or mild cases, which also affects the discriminatory power of the biomarkers. Finally, differences in study design: The clinical cohort employed a 1:1 case–control matching design, which may have amplified the effect size; in contrast, NHANES utilized a cross-sectional natural population design, which more closely resembles real-world screening scenarios.

As a highly heterogeneous multifactorial disorder, the pathogenesis of OSA is considerably complex, primarily involving abnormalities in upper airway anatomy, neuromuscular control dysregulation, respiratory control instability, and alterations in arousal thresholds. These factors collectively lead to partial or complete collapse of the upper airway during sleep, causing recurrent episodes of apnea and hypoventilation (24–28). Among these, upper airway anatomical structures serve as primary aetiological factors. Conditions such as obesity, craniofacial anomalies, and nasal obstruction increase physical airway narrowing and collapse susceptibility (24, 29, 30), thereby elevating pharyngeal critical closing pressure (Pcrit) and heightening obstructive susceptibility during sleep (31, 32). Secondly, neuromuscular dysregulation constitutes another physiological pathogenic factor. Reduced activity of upper airway dilator muscles during sleep, particularly in the REM stage, results in loss of airway tension and subsequent neuro-regulatory deficits (4, 33–35).

Moreover, numerous studies have demonstrated that the pathogenesis of OSA exhibits a considerable degree of correlation with systemic and metabolic factors. Through mechanisms such as intermittent hypoxia, sleep fragmentation, and sympathetic nervous system activation, it significantly elevates the risk of metabolic syndrome, promotes lipid metabolism disorders, and triggers systemic inflammatory responses. This creates a vicious cycle that exacerbates cardiovascular and metabolic diseases (36, 37). Specifically, OSA directly promotes characteristics of metabolic syndrome, including insulin resistance, obesity, and hypertension (38, 39), while simultaneously disrupting lipid metabolism. This manifests as elevated free fatty acids, increased triglycerides, and reduced high-density lipoprotein cholesterol, thereby exacerbating atherosclerosis risk (40–42). Given this pathogenic heterogeneity, traditional single-marker approaches struggle to comprehensively assess individual risk. The novel composite indicator examined in this study, by integrating multidimensional biological information encompassing lipid metabolism, uric acid metabolism, and inflammatory response, better aligns with the systemic disease characteristics of OSA. It offers a novel approach for developing efficient risk assessment tools. At a more microscopic level, the core pathological feature of OSA—intermittent hypoxia/reoxygenation—mimics ischemia–reperfusion injury, inducing oxidative stress and activating NF-κB via ROS as second messengers, thereby initiating and amplifying downstream inflammatory cascades (27, 43). Concurrently, chronic intermittent hypoxia and sleep fragmentation can lead to increased sympathetic nervous system excitability, causing lipid metabolism abnormalities—such as reduced high-density lipoprotein (HDL) and elevated residual cholesterol (RC)—which in turn promote atherosclerosis (44). These processes collectively contribute to changes in indicators such as MHR, AIP, and CMI, making these composite indices potential peripheral biomarkers for OSA-related cardiovascular risk.

Research by Xue Pan, Chen Liao and others has demonstrated that NHHR holds potential as a diagnostic marker for assessing the risk of developing OSA (45, 46); furthermore, relevant studies have also been published concerning CMI, AIP and UHR (47–49). However, reports on RC/HDL, MHR, PHR and SIRI remain relatively scarce. These indices integrate multiple lipid and uric acid metabolism-related markers, demonstrating significant pathophysiological integration. Compared to traditional single markers, they better reflect the overall disease picture and more accurately predict the risk of OSA onset. Specifically, CMI precisely captures the fundamental metabolic context of visceral obesity and insulin resistance; UHR and SIRI quantify metabolic inflammatory burden from the perspectives of oxidative stress-antioxidant balance and systemic immune inflammation, respectively; AIP, RC/HDL, and NHHR directly indicate atherosclerotic risk, reflecting atherogenic lipoprotein characteristics, residual cholesterol risk, and inflammation-lipid interactions respectively; while MHR and PHR further reveal thrombotic inflammatory states and platelet hyperreactivity, linking OSA to acute thrombotic event risk. This indicates that these markers are not only associated with OSA susceptibility but may also predict long-term cardiovascular outcomes at multiple levels. The superior predictive efficacy of markers such as CMI, AIP, RC/HDL, and UHR strongly suggests that atherosclerosis-related inflammatory-lipid interactions and metabolic oxidative stress constitute core components within the pathophysiological network of OSA.

Predictive efficacy diminishes in patients aged over 60 years. From a pathophysiological perspective, this may stem from the fact that in early-onset or younger patients, OSA development is more closely associated with systemic factors such as genetic predisposition, obesity-related metabolic disorders, and chronic inflammation – precisely the core elements reflected by the composite indicators employed in this study. Conversely, in elderly patients, OSA onset may be more predominantly attributable to age-related structural and functional alterations in the upper airway, such as decreased muscle tone, soft tissue laxity, and altered fat distribution—mechanical factors (50). Furthermore, elderly patients in clinical practice frequently present with multiple chronic comorbidities, which themselves can influence lipid, uric acid, and inflammatory marker levels, thereby compromising the specificity of relevant biomarkers. This phenomenon partly explains why all indicators demonstrated superior predictive value in the subgroup without underlying conditions such as hypertension or diabetes. It is worth adding that in this clinical cohort, younger patients exhibited lower predictive efficacy for relevant indicators, potentially related to the age distribution of the attending population. As relatively fewer elderly patients seek clinical treatment, the limited sample size in this age subgroup may have impacted statistical stability. Furthermore, in terms of gender, male patients exhibited significantly higher prevalence than females (51). Moreover, most indicators—except UHR—demonstrated superior efficacy in male patients, consistent with clinical outcomes. This discrepancy may arise because females secrete substantial oestrogen, which effectively promotes HDL secretion and reduces lipid concentrations, thereby introducing certain biases in these indicators.

This study presents several advantages. Firstly, we innovatively adopted a two-stage research design progressing from clinical exploration to validation in a large population cohort: the initial phase established a clinical cohort based on hospital-diagnosed OSA patients, ensuring the accuracy of disease phenotype definition; the subsequent phase utilised the nationally representative NHANES database for independent validation, significantly enhancing the generalisability and extrapolation of the study conclusions. Secondly, the study encompasses both a Chinese clinical cohort and multi-ethnic US national survey data, facilitating preliminary assessment of the applicability and stability of relevant indicators across diverse populations. Moreover, statistical modelling systematically adjusted for confounding factors including demographic characteristics, lifestyle behaviors, and prevalent chronic diseases, supplemented by in-depth stratified analyses to enhance result robustness. Crucially, this study represents the first systematic comparative analysis and associative validation of eight novel inflammatory-metabolic composite indices, providing essential reference for future OSA biomarker screening, evaluation, and translational applications.

Several limitations must nevertheless be acknowledged. Firstly, the primary analysis was based on cross-sectional data; neither the initial clinical cohort nor the NHANES data used for validation could establish a causal relationship between the indicators and OSA. Second, OSA diagnoses in the NHANES database relied on questionnaire surveys rather than the gold standard of polysomnography, which may introduce misclassification bias. The weighted prevalence of OSA symptoms in the NHANES cohort was 43.0%, which is higher than estimates derived from population-based studies. This overestimation reflects the limitations of the questionnaire-based definition: symptoms such as snoring and daytime sleepiness are very common in the general population and may be caused by other factors, such as obesity, insomnia, or upper airway resistance syndrome. The NHANES findings should be interpreted as associations with OSA-related symptoms rather than a diagnosis of OSA. This classification bias dilutes the true association, leading to lower AUC values in the NHANES validation. Finally, despite employing a two-stage analysis strategy—from exploration in a single-centre clinical cohort to validation in a large population database—the limited sample size and single-centre origin of the initial clinical cohort may affect the robustness and representativeness of the preliminary findings.

Future research should prioritise prospective cohort studies confirming OSA diagnosis via polysomnography to clarify the temporal relationships and predictive validity of these indicators. Investigating whether interventions targeting these modifiable metabolic parameters can reduce OSA incidence represents a promising avenue for primary prevention. Finally, integrating the most robust indicators into clinical decision support systems could enhance screening efficiency and implementation feasibility.

## 5 Conclusion

Through a comprehensive analysis of clinical cohorts and a nationally representative survey, this study identified several novel inflammation-metabolic composite indices (including MHR, PHR, NHHR, AIP, UHR, RC/HDL, SIRI, and CMI) that are independently associated with the risk of OSA. These markers integrate multidimensional pathophysiological information spanning lipid metabolism, uric acid homeostasis, and systemic inflammation, and hold potential as adjunctive tools for OSA risk assessment in clinical settings. Further analysis revealed significant subgroup heterogeneity in their predictive performance, with particularly strong performance observed in younger individuals, women, non-obese subjects, and those without severe underlying comorbidities. Overall, these readily available blood-based biomarkers may serve as supplementary indicators for comprehensive OSA risk assessment; however, their clinical application should account for individual characteristics. Nevertheless, differences in individual characteristics must be considered in clinical practice, and prospective studies are needed to validate their predictive value and clinical utility.

## Data Availability

The data that support the findings of this study are available from the corresponding author upon reasonable request. Some data may be subject to privacy or ethical restrictions. Publicly available datasets were analyzed in this study, which can be found at: National Health and Nutrition Examination Survey (https://wwwn.cdc.gov/nchs/nhanes/Default.aspx). Derived data generated in this research are included in this article.
