# Supplementary material for: A novel integrated inflammatory-metabolic indicator as a potential predictor of obstructive sleep apnea: evidence from a clinical cohort and validation in the US National Health and Nutrition Examination Survey
Source: Front Neurol. 2026 Apr 10;17:1813862. doi: 10.3389/fneur.2026.1813862 (PMC13105921; doi:10.3389/fneur.2026.1813862)
Supplement: Supplementary file 1 [file Table_1.DOCX]

**Supplementary Table 1**

ROC parameters of inflammatory-metabolic indices for predicting OSA in clinical cohort

|  | AUC (95% CI) | Optimal cutoff values | Sensitivity | Specificity |
| --- | --- | --- | --- | --- |
| MHR | 0.871(0.832,0.910) | 0.607 | 0.893 | 0.713 |
| PHR | 0.875(0.837,0.913) | 0.580 | 0.860 | 0.720 |
| NHHR | 0.870(0.830,0.909) | 0.593 | 0.853 | 0.740 |
| AIP | 0.874(0.835,0.912) | 0.593 | 0.847 | 0.747 |
| UHR | 0.875(0.837,0.913) | 0.600 | 0.780 | 0.820 |
| RC/HDL | 0.872(0.833,0.910) | 0.587 | 0.853 | 0.733 |
| SIRI | 0.867(0.827,0.907) | 0.593 | 0.880 | 0.713 |

**Abbreviations:** OSA, obstructive sleep apnea; CI, confidence intervals; MHR, Monocyte to HDL Ratio; PHR, Platelet to HDL Ratio; NHHR, Non-HDL to HDL Ratio; AIP, Atherogenic Index of Plasma; UHR, Uric acid to HDL Ratio; RC/HDL, Remnant Cholesterol to HDL Ratio.
