# Supplementary material for: A novel integrated inflammatory-metabolic indicator as a potential predictor of obstructive sleep apnea: evidence from a clinical cohort and validation in the US National Health and Nutrition Examination Survey
Source: Front Neurol. 2026 Apr 10;17:1813862. doi: 10.3389/fneur.2026.1813862 (PMC13105921; doi:10.3389/fneur.2026.1813862)
Supplement: Supplementary file 2 [file Table_2.DOCX]

**Supplementary Table 2**

ROC parameters of inflammatory-metabolic indices for predicting OSA in NHANES cohort

|  | AUC (95% CI) | Optimal cutoff values | Sensitivity | Specificity |
| --- | --- | --- | --- | --- |
| MHR | 0.572(0.555,0.590) | 0.126 | 0.421 | 0.706 |
| PHR | 0.564(0.547,0.582) | 0.132 | 0.655 | 0.477 |
| NHHR | 0.577(0.560,0.594) | 0.155 | 0.742 | 0.413 |
| AIP | 0.585(0.569,0.603) | 0.170 | 0.722 | 0.448 |
| UHR | 0.597(0.580,0.614) | 0.164 | 0.450 | 0.714 |
| RC/HDL | 0.586(0.569,0.603) | 0.175 | 0.721 | 0.454 |
| SIRI | 0.520(0.503,0.537) | 0.078 | 0.741 | 0.337 |
| CMI | 0.603(0.586,0.620) | 0.211 | 0.724 | 0.486 |

**Abbreviations:** OSA, obstructive sleep apnea; CI, confidence intervals; MHR, Monocyte to HDL Ratio; PHR, Platelet to HDL Ratio; NHHR, Non-HDL to HDL Ratio; AIP, Atherogenic Index of Plasma; UHR, Uric acid to HDL Ratio; RC/HDL, Remnant Cholesterol to HDL Ratio; SIRI, Systemic Inflammation Response Index.
