# Supplementary material for: A novel integrated inflammatory-metabolic indicator as a potential predictor of obstructive sleep apnea: evidence from a clinical cohort and validation in the US National Health and Nutrition Examination Survey
Source: Front Neurol. 2026 Apr 10;17:1813862. doi: 10.3389/fneur.2026.1813862 (PMC13105921; doi:10.3389/fneur.2026.1813862)
Supplement: Supplementary file 3 [file Table_3.DOCX]

**Supplementary Table 3**

Unweighted Multivariable Logistic Regression Analysis of Multiple serum composite indicators and Obstructive Sleep Apnea

|  | Model Ⅰ  OR (95% CI) | P-value | Model Ⅱ  OR (95% CI) | P-value | Model Ⅲ  OR (95% CI) | P-value |
| --- | --- | --- | --- | --- | --- | --- |
| MHR | | | | | | |
| Q1  0.04-0.28 | Ref |  | Ref |  | Ref |  |
| Q2  0.28-0.38 | 1.26(1.06,1.50) | 0.008 | 1.22(1.03,1.46) | 0.025 | 1.04(0.87,1.26) | 0.643 |
| Q3  0.38-0.52 | 1.38(1.16,1.64) | <0.001 | 1.30(1.09,1.56) | 0.004 | 1.01(0.83,1.21) | 0.956 |
| Q4  0.52-3.67 | 1.99(1.67,2.37) | <0.001 | 1.80(1.50,2.17) | <0.001 | 1.27(1.04,1.55) | 0.021 |
| PHR | | | | | | |
| Q1  30.06-135.82 | Ref |  | Ref |  | Ref |  |
| Q2  135.82-179.28 | 1.26(1.06,1.50) | 0.010 | 1.26(1.06,1.51) | 0.010 | 1.13(0.94,1.36) | 0.195 |
| Q3  179.28-231.67 | 1.49(1.25,1.77) | <0.001 | 1.54(1.29,1.84) | <0.001 | 1.20(1.00,1.45) | 0.052 |
| Q4  231.67-817.33 | 1.83(1.54,2.17) | <0.001 | 1.99(1.66,2.38) | <0.001 | 1.39(1.15,1.68) | <0.001 |
| NHHR | | | | | | |
| Q1  0.28-1.80 | Ref |  | Ref |  | Ref |  |
| Q2  1.80-2.47 | 1.55(1.30,1.85) | <0.001 | 1.54(1.29,1.84) | <0.001 | 1.35(1.13,1.63) | 0.001 |
| Q3  2.47-3.31 | 1.74(1.46,2.08) | <0.001 | 1.66(1.39,1.99) | <0.001 | 1.35(1.12,1.63) | 0.002 |
| Q4  3.31-9.26 | 2.10(1.76,2.50) | <0.001 | 1.98(1.65,2.38) | <0.001 | 1.46(1.20,1.77) | <0.001 |
| AIP | | | | | | |
| Q1  (-1.25)-(-0.32) | Ref |  | Ref |  | Ref |  |
| Q2  (-0.32)-(-0.11) | 1.44(1.20,1.71) | <0.001 | 1.37(1.15,1.64) | <0.001 | 1.16(0.96,1.39) | 0.130 |
| Q3  (-0.11)-0.13 | 2.00(1.68,2.39) | <0.001 | 1.90(1.58,2.27) | <0.001 | 1.38(1.14,1.67) | <0.001 |
| Q4  0.13-0.86 | 2.14(1.80,2.55) | <0.001 | 2.03(1.69,2.44) | <0.001 | 1.32(1.09,1.62) | 0.005 |
| UHR | | | | | | |
| Q1  0.01-0.07 | Ref |  | Ref |  | Ref |  |
| Q2  0.07-0.10 | 1.59(1.33,1.90) | <0.001 | 1.52(1.27,1.82) | <0.001 | 1.19(0.99,1.44) | 0.069 |
| Q3  0.10-0.14 | 1.79(1.50,2.13) | <0.001 | 1.65(1.37,1.98) | 0.001 | 1.14(0.93,1.39) | 0.199 |
| Q4  0.14-0.43 | 2.51(2.11,3.00) | <0.001 | 2.22(1.83,2.70) | <0.001 | 1.28(1.03,1.60) | 0.024 |
| RC/HDL | | | | | | |
| Q1  0.03-0.22 | Ref |  | Ref |  | Ref |  |
| Q2  0.22-0.36 | 1.43(1.20,1.70) | <0.001 | 1.36(1.14,1.63) | <0.001 | 1.15(0.96,1.39) | 0.135 |
| Q3  0.36-0.62 | 2.04(1.71,2.43) | <0.001 | 1.93(1.61,2.32) | <0.001 | 1.40(1.16,1.70) | <0.001 |
| Q4  0.62-3.29 | 2.16(1.81,2.58) | <0.001 | 2.05(1.71,2.47) | <0.001 | 1.35(1.11,1.65) | 0.003 |
| SIRI | | | | | | |
| Q1  0.08-0.64 | Ref |  | Ref |  | Ref |  |
| Q2  0.64-0.97 | 1.14(0.96,1.35) | 0.145 | 1.12(0.94,1.34) | 0.196 | 1.44(0.87,1.25) | 0.656 |
| Q3  0.97-1.46 | 1.22(1.03,1.45) | 0.022 | 1.20(1.00,1.43) | 0.045 | 1.04(0.87,1.25) | 0.667 |
| Q4  1.46-11.60 | 1.18(0.99,1.40) | 0.057 | 1.12(0.94,1.35) | 0.205 | 0.96(0.79,1.16) | 0.637 |
| CMI | | | | | | |
| Q1  0.03-0.27 | Ref |  | Ref |  | Ref |  |
| Q2  0.27-0.48 | 1.45(1.22,1.73) | <0.001 | 1.38(1.15,1.65) | <0.001 | 1.07(0.89,1.29) | 0.483 |
| Q3  0.48-0.86 | 2.27(1.90,2.70) | <0.001 | 2.13(1.78,2.56) | <0.001 | 1.40(1.15,1.70) | <0.001 |
| Q4  0.86-5.22 | 2.48(2.08,2.97) | <0.001 | 2.39(1.98,1.87) | <0.001 | 1.32(1.07,1.63) | 0.010 |

Model Ⅰ: unadjusted model.

Model Ⅱ: adjusted for age, gender, race, education level, Marital, PIR.

Model Ⅲ:adjusted for variables in Model Ⅱ plus drinking status, BMI, hypertension, diabetes status, CVD, physical activity level, smoking status;

Abbreviations: BMI, body mass index; PIR: family income-poverty ratio; MHR, Monocyte to HDL Ratio; PHR, Platelet to HDL Ratio; NHHR, Non-HDL to HDL Ratio; AIP, Atherogenic Index of Plasma; UHR, Uric acid to HDL Ratio; RC/HDL, Remnant Cholesterol to HDL Ratio; SIRI, Systemic Inflammation Response Index; CMI, Cardiometabolic Index; CVD, cardiovascular disease; OR, odds ratio; 95% CI, 95% confidence interval.
